# Expression of *Bacillus thuringiensis* toxin Cyt2Ba in the entomopathogenic fungus *Beauveria bassiana* increases its virulence towards *Aedes* mosquitoes

**DOI:** 10.1371/journal.pntd.0007590

**Published:** 2019-07-15

**Authors:** Sheng-Qun Deng, Wei-Hao Zou, Dong-Liang Li, Jia-Ting Chen, Qiang Huang, Li-Juan Zhou, Xiao-Xue Tian, Yi-Jun Chen, Hong-Juan Peng

**Affiliations:** Department of Pathogen Biology, Guangdong Provincial Key Laboratory of Tropical Disease Research, Southern Medical University, Guangzhou, Guangdong Province, China; Faculty of Science, Mahidol University, THAILAND

## Abstract

**Background:**

The entomopathogenic fungus *Beauveria bassiana* has been widely used to kill mosquito larvae and adults in the laboratory and field. However, its slow action of killing has hampered its widespread application. In our study, the *B*. *bassiana* fungus was genetically modified to express the *Bacillus thuringiensis (Bt)* toxin Cyt2Ba to improve its efficacy in killing mosquitoes.

**Methodology/Principal findings:**

The efficacy of the wild type (WT) of *B*. *bassiana* and a transgenic strain expressing Cyt2Ba toxin (*Bb*-Cyt2Ba) was evaluated against larval and adult *Aedes* mosquitoes (*Aedes aegypti* and *Aedes albopictus*) using insect bioassays. The *Bb*-Cyt2Ba displayed increased virulence against larval and adult *Aedes* mosquitoes compared with the WT: for *Ae*. *aegypti* adults, the median lethal time (LT_50_) was decreased by 33% at the concentration of 1× 10^8^ conidia/ml, 19% at 1× 10^7^ conidia/ml and 47% at 1× 10^6^ conidia/ml. The LT_50_ for *Ae*. *albopictus* adults was reduced by 20%, 23% and 29% at the same concentrations, respectively. The LT_50_ for *Ae*. *aegypti* larvae was decreased by 42% at 1× 10^7^ conidia/ml and 25% at 1× 10^6^ conidia/ml, and that for *Ae*. *albopictus* larvae was reduced by 33% and 31% at the same concentrations, respectively. In addition, infection with *Bb*-Cyt2Ba resulted in a dramatic reduction in the fecundity of *Aedes* mosquitoes.

**Conclusions/Significance:**

In conclusion, our study demonstrated that the virulence of *B*. *bassiana* against mosquitoes can be significantly improved by introducing the *Bt* toxin gene *Cyt2Ba* into the genome to express the exogenous toxin in the fungus. The transgenic strain *Bb*-Cyt2Ba significantly reduced the survival and fecundity of *Ae*. *aegypti* and *Ae*. *albopictus* compared with the WT strain, which suggested that this recombinant *B*. *bassiana* has great potential for use in mosquito control.

## Author summary

Mosquito vectors transmit many diseases to humans and animals, causing illness and death and resulting in substantial socio-economic burdens in endemic countries. The control of mosquitoes has almost exclusively relied on the use of chemical insecticides, which has recently led to the broad resistance of important mosquito vectors worldwide. Entomopathogenic fungi, such as *Beauveria bassiana*, are an important alternative or complement to chemical insecticides. However, the relatively slow action of fungal pathogens in killing mosquitoes, compared with chemical insecticides, has hampered their widespread application. To improve the insecticidal efficacy of the entomopathogen *B*. *bassiana*, the fungus was genetically modified to express the *Bacillus thuringiensis* toxin Cyt2Ba. The mitotically stable transformant (*Bb*-Cyt2Ba) successfully expressed Cyt2Ba toxin, and the virulence of this strain against adults and larvae of *Aedes aegypti* and *Aedes albopictus* mosquitoes was significantly improved. In addition, egg laying was significantly affected by *Bb*-Cyt2Ba infection. Infection with this fungus resulted in a dramatic reduction in fecundity of the target mosquitoes. Therefore, this recombinant *B*. *bassiana* has great potential for use in mosquito control.

## Introduction

Mosquito vectors transmit many diseases to humans and animals, causing illness and death that result in considerable socio-economic burdens in endemic countries [1]. *Aedes* mosquitoes (primarily *Aedes aegypti* and *Aedes albopictus*) are the primary vectors of dengue, Zika, chikungunya and yellow fever in tropical and subtropical zones, which have a devastating impact on human health [2, 3]. Vector control via chemical insecticides is a major method for vector-borne disease control, but the extensive use of chemical insecticides poses toxicity risks to humans as well as the environment and creates intensive pressure for mosquitoes to develop resistance [4].

Biological control agents such as entomopathogenic fungi are important alternatives or complements to chemical insecticides [5]. Many studies have shown the potential of entomopathogenic fungi, such as *Beauveria bassiana*, for the control of agricultural pests [6] and the vectors of human diseases, including mosquitoes [7]. One of the main advantages of using entomopathogenic fungi in insect control is that these fungi can infect all stages of the insects, including larvae and adults [8–10]. Furthermore, no cases of resistance of insects to entomopathogenic fungal infections have been reported to date [11]. However, the relatively slow action of fungal pathogens in killing vectors, compared with chemical insecticides, has hampered their widespread application in the field.

Genetic engineering is essential for introducing desirable traits into entomopathogenic fungi [12], which has resulted in a wide range of feasible types of genetic manipulation, from the expression of UV protectants, heat shock factors, immune modulators, cuticle-degrading enzymes and exogenous toxins to targeting insect vectors, disease transmission, and even arthropod behaviors [12]. It has been reported that transgenic *B*. *bassiana* expressing *Ae*. *aegypti* trypsin modulating oostatic factor (TMOF) exhibited increased virulence against *Anopheles gambiae* compared with the wild type strain [13]. Our previous study also found that the expression of the insect-specific toxin scorpion neurotoxin AaIT in *B*. *bassiana* enhanced virulence against *Ae*. *albopictus* mosquitoes [14]. The virulence of *B*. *bassiana* for mosquitoes could be increased via the expression of insecticidal toxins.

*Bacillus thuringiensis (Bt)*, an entomopathogenic bacterium, is also an important bioinsecticide used for control of insects, including mosquitoes [15]. Based on the last updated data in the especial database for *Bt* toxins (June 2018), about 323 holotype toxins have been identified and characterized in the *Bt* strains isolated from different regions of the world [16]. The cytolytic δ-endotoxin (Cyt2Ba) containing 263 amino acids is found at very low concentrations in *Bt* crystals [17]. It has been reported that Cyt2Ba is toxic to *Culex*, *Aedes* and *Anopheles* larvae [18, 19]. In our study, the *Cyt2Ba* gene was introduced into the *B*. *bassiana* genome by genetic engineering to improve fungal virulence. We measured the virulence of this recombinant *B*. *bassiana* to the adults and larvae of *Aedes* mosquitoes (*Ae*. *aegypti* and *Ae*. *albopictus*). The effect of this strain on mosquito fecundity was also determined.

## Methods

### Ethics statement

Our only experimental animals are *Aedes aegypti* and *Aedes albopictus*, which do not involve animal ethical issues.

### Mosquitoes

The Guangdong Provincial Center for Disease Control and Prevention collected *Ae*. *albopictus* and *Ae*. *aegypti* mosquitoes from different sites in the cities of Foshan and Zhanjiang in Guangdong Province. All mosquitoes were reared in standard insectary conditions at (28 ± 1) °C and (80 ± 5) % relative humidity with a light:dark cycle of 16 h:8 h. The larvae were fed daily with turtle food (Shenzhen INCH-GOLD Fish Food,.LTD, Shenzhen, CHA). Mosquito adults were provided with 10% glucose solution ad libitum. All collection was done on public land.

### Microbial strains and media

The *B*. *bassiana* GIM3.428 strain (wild type) was purchased from the Guangdong Microbiology Culture Center and maintained on Czapek’s agar (CDA) at 4°C for preservation. Conidia were obtained by growing the fungus on CDA for 7 days at 25°C. Blastospores were obtained by the growth of the fungus in Sabouraud dextrose broth for 48 h and glucose-mineral medium for 24 h at 25°C under 120 rpm on a rotatory shaker.

### Gene synthesis and vector construction

The coding sequence of Cyt2Ba (GenBank: GQ919041.1) was synthesized (Generay Biotech, Shanghai, CHA) with the *B*. *bassiana* preferred coding usage and cloned between the *BamH*I and *EcoR*I sites of pBARGPE1 to generate the plasmid pBARGPE1-Cyt2Ba. This plasmid retains a strong gpdA promoter to drive the insert’s gene expression and has the phosphinothricin (PPT) resistance gene (*Bar*) as a selectable marker for fungal transformation.

### Fungal transformation

The plasmid pBARGPE1-Cyt2Ba was linearized with *Sca*I and then transformed into *B*.*bassiana* blastospores by electroporation as described previously [14]. Transformants were grown on CDA plates containing 150 μg/ml PPT at 25°C. After single spore isolation and subsequent subculturing for three generations on CDA with 150 μg/ml PPT at 25°C (7 days each generation), the putative transformants were verified by polymerase chain reaction (PCR) using primers for the *bar* gene (Bar-F, TCGTCAACCACTACATCGAGAC and Bar-R, GAAGTCCAGCTGCCAGAAAC) and *Cyt2Ba* gene (Cyt-F TATGGATCCGCCACCATGGAAC, and Cyt-R, TATGAATTCCTAGGACTTGATGGG). Furthermore, to verify the mitotic stability of their PPT resistance, the recombinants were subcultured for three generations on CDA without PPT at 25°C, and finally, they were subcultured on CDA with 400 μg/ml PPT at 25°C. Then, the transformants were analyzed by PCR. A stable transformant named *Bb*-Cyt2Ba was selected for the subsequent experiments.

### Identification of Cyt2Ba expression in *Bb*-Cyt2Ba

The wild type (WT) and *Bb*-Cyt2Ba strains were grown on CDA for 4 days. The conidia were collected with cotton swabs. Mosquitoes were infected by contact with WT or *Bb*-Cyt2Ba conidia on the swab. Dead female adult *Ae*. *albopictus* mosquitoes were maintained at 25°C under saturated humidity for 4 days. To verify the transcription of the Cyt2Ba gene, total RNA was extracted from the CDA culture supernatant or infected mosquitoes by using an RNeasy mini plant kit (Qiagen, Duesseldorf, GER). Reverse-transcription PCR (RT-PCR) was performed using the primer pair Cyt-F and Cyt-R.

The expression of Cyt2Ba was detected by western blot analyses. Cyt2Ba polyclonal antibodies were raised in rabbits (BGI Genomics, Shenzhen, CHA), and an alkaline phosphatase-conjugated anti-rabbit IgG secondary antibody (Santa Cruz Biotech, Newport, USA) was used for detection. The total proteins were extracted from the CDA culture supernatant and the infected mosquitoes as previously described (14). Fifty micrograms of each sample was separated on a 12% polyacrylamide gel by sodium dodecyl sulfate (SDS) polyacrylamide gel electrophoresis, and western blotting was then performed.

### Survival assays

*B*. *bassiana* strains were grown and maintained on CDA at 25°C. Conidial suspensions in 0.02% (vol/vol) Tween 80 were prepared from 7-d-old cultures. To conduct fungal infection, adult female *Aedes* mosquitoes were transferred to the filter (placed on a 300 ml plastic cup covered with a net), which had absorbed 10 ml of conidial suspension at 1 × 10^6^ conidia/ml (low), 1 × 10^7^ conidia/ml (middle) or 1 × 10^8^ conidia/ml (high) or 0.02% Tween 80 (control) for 3 h. Then, the mosquitoes were transferred separately to different plastic containers (30 mosquitoes/cup). The contaminated or control mosquitoes were maintained on a 10% glucose diet in plastic containers at 28°C. The dead adult mosquitoes in each treatment group were counted and removed every 12 h until the last mosquito died. The dead mosquitoes were washed twice in phosphate-buffered saline and placed in humid filter papers for conidiation. To infect the larvae, conidial suspensions of 1 × 10^5^ conidia/ml (low), 1 × 10^6^ conidia/ml (middle) and 1 × 10^7^ conidia/ml (high) of *B*. *bassiana* were added to the cups containing 30 second-instar *Aedes* larvae in 30 ml of double-distilled water (ddH_2_O). Thirty individuals in ddH_2_O without fungi were used as the control. The larvae were provided with turtle food at a rate of 0.2–0.3 mg/larva per day and maintained at 28°C. The larvae were monitored every 12 h for survival (the percentage surviving) until day 10. Each treatment for the larvae or adults contained 90 (30 × 3) mosquitoes. The survival of the adults or larvae in each group was calculated every day. The bioassay was repeated three times for statistical analysis.

### The effects of wild type or recombinant fungus infection on mosquito fecundity

To examine the effect of fungal infection on the fecundity of mosquitoes, *Aedes* females were infected by contact with the filter paper that had absorbed 10 ml of the conidial suspension with 1 × 10^8^ conidia/ml of the wild type or *Bb*-Cyt2Ba strain. After fungal infection, the females were fed with defibrinated sheep blood for 1 h. Then, the engorged mosquitoes were placed individually into a 250-ml, gauze-covered paper cup containing a small amount of water and funnel-shaped filter paper to serve as a repository for the eggs. The eggs of each mosquito were counted one week after blood feeding. Sixty female mosquitoes per treatment were used to assess the impact of fungal infection on fecundity.

### Statistical analysis

For survival assays, data were analyzed using Probit and Kaplan–Meier survival tests; a log-rank test was used to assess the equality of survival distributions during the Kaplan–Meier analysis. Furthermore, cross-tabulation analyses using Pearson’s chi-square test were applied to compare the percentages of ovipositing females among the different groups. In addition, Mann-Whitney tests were applied to compare the fecundity among the groups. All analyses were performed with SPSS (v. 20.0), and significance was defined by *P*<0.05.

## Results

### The *Cyt2Ba* gene was stably inherited in the *Bb*-Cyt2Ba strain

Transformation of the competent blastopores of *B*. *bassiana* with linearized plasmid pBARGPE1-Cyt2Ba produced one transgenic colony on the CDA plate containing 150 μg/ml PPT. The transformant was able to grow on a CDA plate containing 150 μg/ml PPT for three generations. Then, after three rounds of subculturing on PPT-free CDA plates, the transformant was capable of growing on the CDA plate containing 400 μg/ml PPT. The expected PCR fragments from all the *Bb*-Cyt2Ba samples appeared on the agarose gel, but not the WT (S1 Fig), which confirmed the consistent heredity of the *Cyt2Ba* gene in the *Bb*-Cyt2Ba genome. Transformation of the pBARGPE1-Cyt2Ba construct into *B*. *bassiana* did not affect the germination rate of the fungus after 24 h, and we did not find any morphological differences between the germ tubes of WT and *Bb*-Cyt2Ba.

### Cyt2Ba toxin was stably expressed in the *Bb*-Cyt2Ba strain

**Fig 1:**
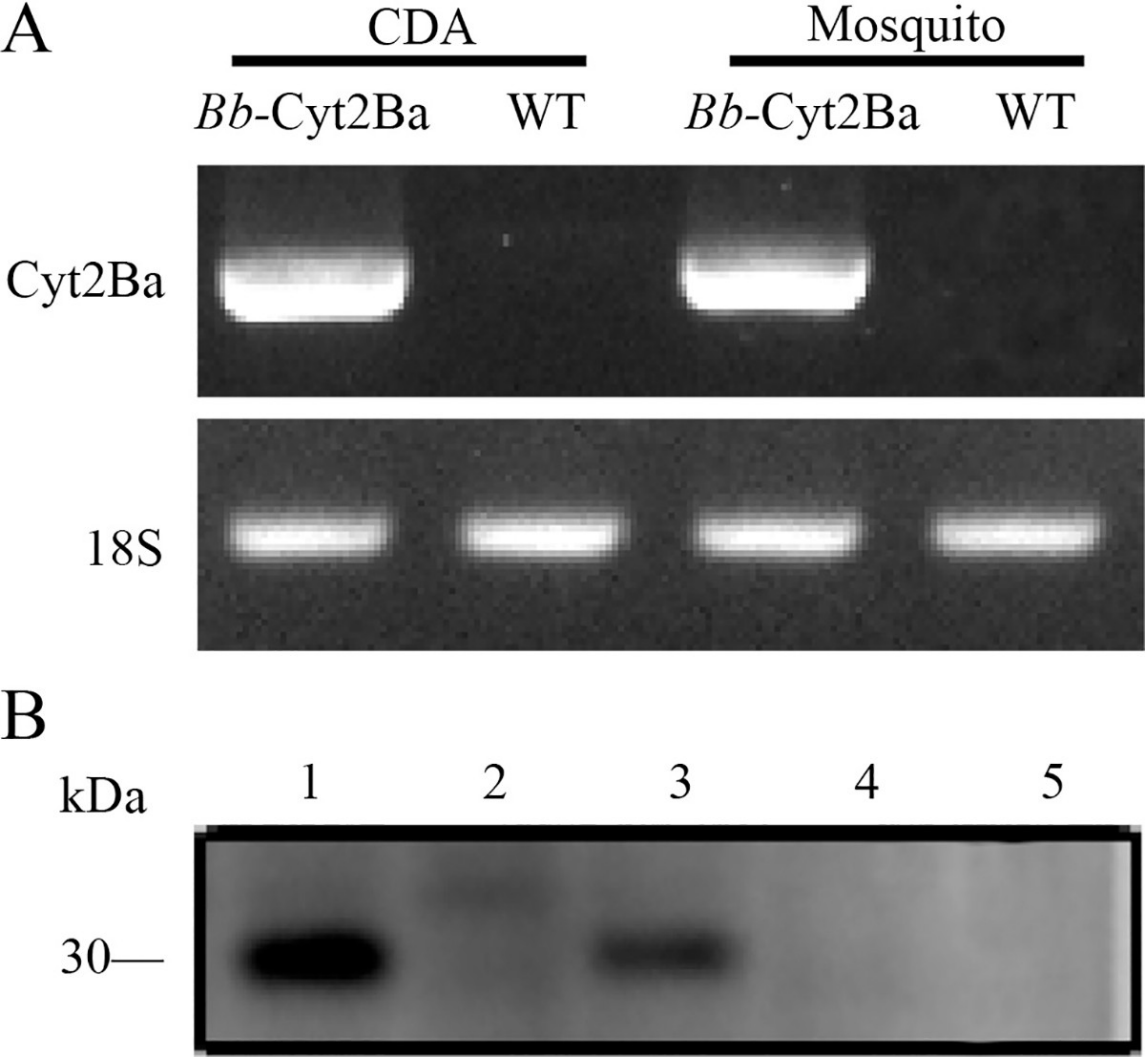
Evidence for Cyt2Ba expression in the *Bb*-Cyt2Ba strain. (A) RT-PCR detection of *Cyt2Ba* gene transcription in *Bb*-Cyt2Ba collected from the infected dead female *Ae*. *albopictus* mosquitoes and the CDA with positive results and in the WT control with negative results. The 18S rRNA gene was detected for the loading control. (B) Western blot detection of Cyt2Ba expression in *Bb*-Cyt2Ba and the WT with a polyclonal antibody against Cyt2Ba from different samples. Lane 1, culture supernatant from *Bb*-Cyt2Ba grown in CDA for 4 days with a positive result; Lane 2, culture supernatant from WT grown in CDA for 4 days with a negative result; and Lane 3, the dead female *Ae*. *albopictus* mosquitoes infected by *Bb*-Cyt2Ba and incubated at 25°C for another 4 days; Lane 4, the dead female mosquitoes infected by the WT and incubated at 25°C for another 4 days; Lane 5, loading control.

The WT and *Bb*-Cyt2Ba strains were harvested from the CDA culture after incubation for 4 days at 25°C. The fungal outgrowths were those of a typical *B*. *bassiana*, and they appeared on the dead female *Aedes* mosquitoes that were infected through cuticle penetration after being maintained at 25°C in saturated humidity for 4 days. RT-PCR and western blot analyses confirmed that the recombinant strain expressed the Cyt2Ba toxin stably, but not the WT strain (Fig 1).

### The survival of mosquitoes was reduced by *Bb*-Cyt2Ba infection

**Fig 2:**
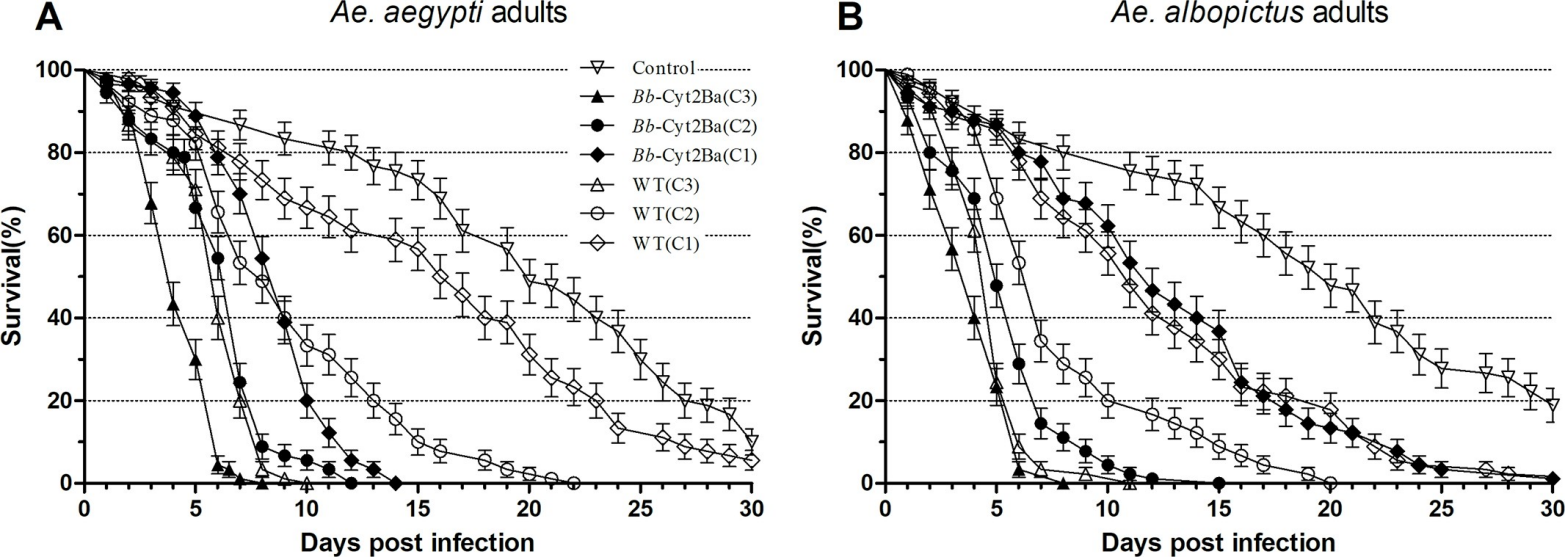
Survival curves of the adult mosquitoes for different *Bb*-Cyt2Ba and WT treatments. The survival curves of *Ae*. *aegypti* (A) and *Ae*. *albopictus* (B) females when treated with (C1) 1 × 10^6^, (C2) 1 × 10^7^ and (C3) 1 × 10^8^ conidia/ml suspensions of *Bb*-Cyt2Ba and WT. Each treatment contained 90 mosquitoes, performed three times.

**Fig 3:**
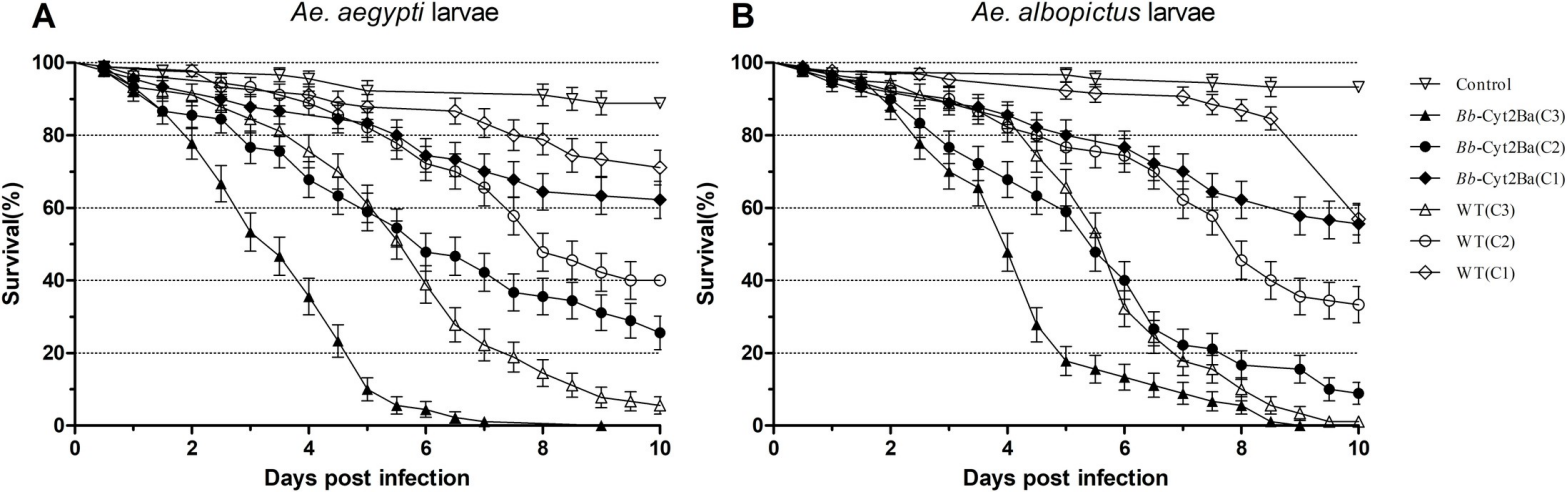
Survival curves of the mosquito larvae for different *Bb*-Cyt2Ba and WT treatments.

The mosquito mortalities generally increased with the elevated conidial concentration and prolonged post-treatment time for both *Bb*-Cyt2Ba and *Bb*-WT (Figs 2 and 3). Significant differences were found among the groups treated with different concentrations of *Bb*-Cyt2Ba or the *Bb*-WT (S1 Table). In addition, significant differences were found between the mortalities of *Bb*-Cyt2Ba and the WT-treated *Aedes* adults at each concentration (S2 Table). Thus, the adult mosquitoes that were treated with *Bb*-Cyt2Ba tended to die faster than those treated with the WT at the same concentration (Fig 2), and the difference was significant (S2 Table). Similarly, except for *Ae*. *aegypti* larvae infected with fungi at the low concentration, *Bb*-Cyt2Ba-treated larvae tended to die faster than those treated with the WT at each concentration (Fig 3), and the difference was significant (S2 Table).

The survival curves of *Ae*. *aegypti* (A) and *Ae*. *albopictus* (B) females when treated with (C1) 1 × 10^5^, (C2) 1 × 10^6^ and (C3) 1 × 10^7^ conidia/ml suspensions of *Bb*-Cyt2Ba and WT. Each treatment contained 90 larvae, performed three times. The LT_50_s of the female *Ae*. *aegypti* for *Bb*-Cyt2Ba treatment were reduced by 33%, 19% and 47% compared with those for WT treatment at the high, middle and low concentrations, respectively (Table 1). Moreover, the LT_50_s of *Ae*. *albopictus* adults for *Bb*-Cyt2Ba treatment were reduced by 20%, 23% and 29% at these three concentrations compared with those for WT treatment. In addition, compared with the WT, the LT_50_ of the *Bb*-Cyt2Ba infection was reduced by 42% and 25% for *Ae. aegypti* larvae and 33% and 31% for *Ae. albopictus* larvae at the high and middle concentrations, respectively (Table 1). These results indicated that the expressed Cyt2Ba toxin in the fungus reduced the survival of the tested larvae and adult mosquitoes.

**Table 1:** The median lethal times (LT_50_s) of the fungal strains *Bb*-Cyt2Ba and WT against *Aedes* mosquitoes (larvae or adults) at three concentrations.

Mosquitoes	Fungal strains	LT_50_ (± standard error), day		
	(conidia/ml)	1 x 10^8^	1 x 10^7^	1 x 10^6^
*Ae*. *aegypti* adults	*Bb*-Cyt2Ba	4.0 (±0.2)	6.5 (±0.2)	8.5 (±0.3)
	WT	6.0 (±0.1)	8.0 (±0.5)	16.0 (±1.3)
*Ae*. *aegypti* larvae	*Bb*-Cyt2Ba		3.5 (±0.3)	6.0 (±0.7)
	WT		6.0 (±0.2)	8.0 (±0.5)
*Ae. albopictus* adults	*Bb*-Cyt2Ba	4.0 (±0.3)	5.0 (±0.3)	10.0 (±0.5)
	WT	5.0 (±0.3)	6.5 (±0.3)	14.0 (±1.7)
*Ae. albopictus* larvae	*Bb*-Cyt2Ba		4.0 (±0.1)	5.5 (±0.1)
	WT		6.0 (±0.3)	8.0 (±0.3)

The difference in the LT_50_ between the *Bb*-Cyt2Ba and the WT strains at each concentration shown above is significant.

### The fecundity of female mosquitoes was reduced by *Bb*-Cyt2Ba infection

Egg laying was significantly affected by fungal infection. The percentage of *Bb*-Cyt2Ba-infected *Ae*. *aegypti* females that oviposited within 7 days after a blood meal was only 45% (27/60), which was significantly lower compared to that of the WT (65%, 39/60) and the control (95%, 57/60) (S3 Table). Similarly, the percentage of *Ae*. *albopictus* mosquitoes producing eggs was 50% (30/60), which was significantly lower than that of the WT (70%, 42/60) and the control (97%, 58/60) (S3 Table).

**Fig 4:**
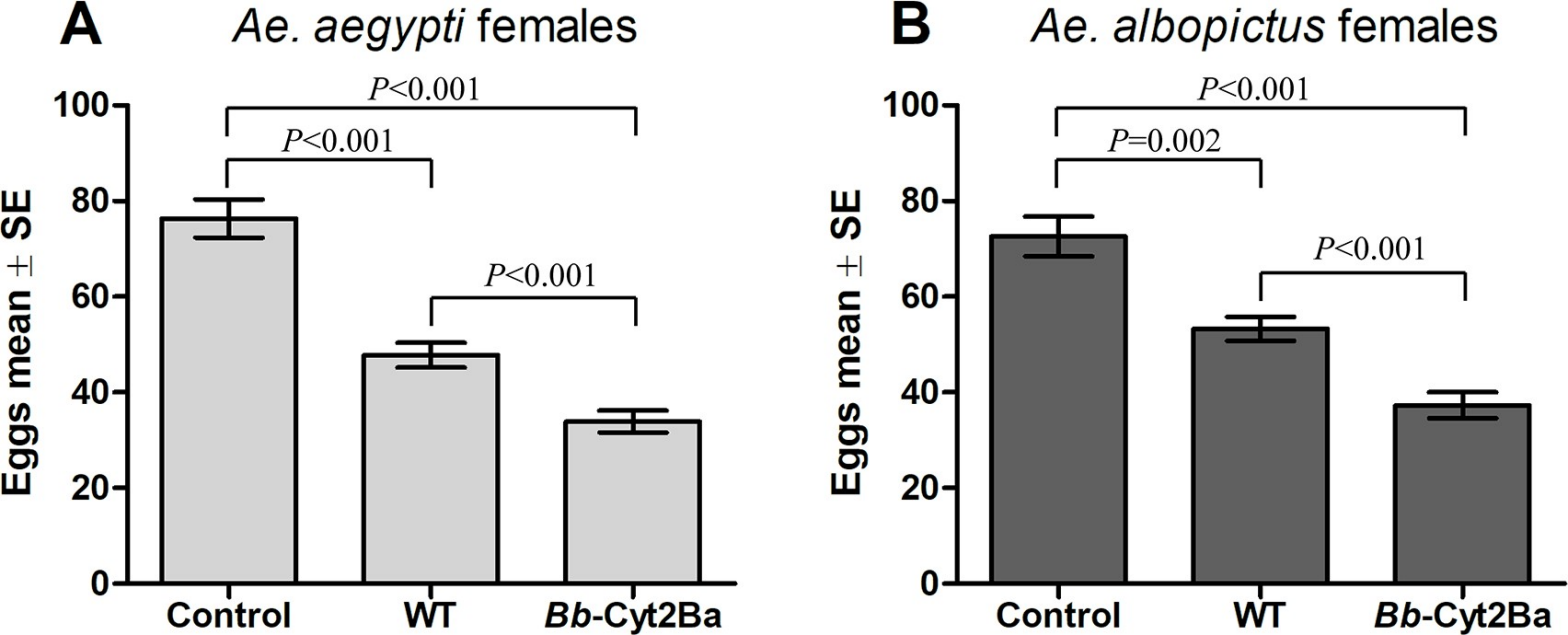
Effects of *Bb*-Cyt2Ba infection on mosquito reproduction. *Ae*. *aegypti* (A) and *Ae*. *albopictus* (B) females infected with a suspension of 1 × 10^8^ conidia/ml of the WT or *Bb*-Cyt2Ba strain were fed a blood meal 3 h after fungal infection. Noninfected, blood-fed mosquitoes were used as controls. Each bar represents the mean of eggs per mosquito. Mann-Whitney tests were used. Error bars indicate ± standard error (SE).

The mean number of eggs produced per female within 7 days for *Ae*. *aegypti* was 33.9 (95% CI: 29.2–38.6) in the *Bb*-Cyt2Ba-treated group, 47.8 (95% CI: 42.5–53.1) in the WT-treated group and 76.4 (95% CI: 68.4–84.4) in the control group (Fig 4A). For *Ae*. *albopictus* mosquitoes, the mean number of eggs produced per female was 37.3 (95% CI: 31.8–42.8) in the *Bb*-Cyt2Ba-treated group, 53.3 (95% CI: 48.2–58.4) in the WT-treated group and 72.6 (95% CI: 64.3–81.0) in the control group (Fig 4B). Thus, infection of mosquitoes with the wild type strain resulted in a significant reduction (37% for *Ae*. *aegypti* and 27% for *Ae*. *albopictus*) in fecundity compared to noninfected controls. However, expression of Cyt2Ba toxin in the fungi resulted in a dramatic reduction (56% for *Ae*. *aegypti*, 49% for *Ae*. *albopictus*) in fecundity compared to the controls.

## Discussion

The slow action of insect-pathogenic fungi on target pests is one of the limitations to their commercialization and large-scale application as biocontrol agents [4]. Some studies have shown that the insect-killing efficacy of *B*. *bassiana* is considerably improved by recombination with the exogenous *Ae*. *aegypti* TMOF gene, scorpion neurotoxin AaIT gene [8, 20], cuticle degradation protease PR1A gene [21], or vegetative insecticidal proteins of *Bacillus thuringiensis* (*Bt*) [22].

Cytolytic (Cyt) toxins are produced by a minor group of *Bt*, mostly in subspecies that are toxic to Dipteran insects [23, 24]. These toxins do not need a specific receptor but directly interact with membrane lipids and insert into the membrane to form pores [25–27] or destroy the membrane by a detergent-like interaction [28]. In our study, the cytolytic toxin Cyt2Ba gene was genetically introduced into the genome of the wild type of *B*. *bassiana* to enhance its virulence against mosquitoes. The mitotically stable transformant *Bb*-Cyt2Ba can successfully express this exogenous toxin, and the virulence of *Bb*-Cyt2Ba for *Ae*. *aegypti* and *Ae*. *albopictus* mosquitoes (including adults and larvae) was significantly increased compared with that of the WT.

When adult mosquitoes were infected by the fungus through cuticle contact, the fungus quickly invaded the mosquito tissues and cells. It has been reported that approximately 24 h was required for the fungus to penetrate the cuticle and reach the hemocoel of insects [29, 30]. In our study, the fungus may have released Cyt2Ba toxin into the mosquito hemocoel after penetration, which resulted in quicker death for the *Aedes* adult mosquitoes. Furthermore, it has been reported that when fungal spores are applied to an aquatic habitat, typical for mosquito larvae, the nutrients in the water are usually sufficient to stimulate the germination of the spores following water intake [31, 32]. For mosquito larvae, the main infection routes are through feeding and respiration [33]. The Cyt2Ba toxin is supposed to be delivered to the insect circulatory system after *Bb*-Cyt2Ba infects the mosquito larvae, which results in faster death of the larvae compared with the WT infection. The various commercial products of *Bt* have been used in the control of mosquito larvae. Formulations include a variety of granules, flowable concentrates, wettable powders, and slow-release tablets and briquettes [34]. The efficacy of *Bt* formulations has been demonstrated in a diversity of habitats against a multitude of species of mosquitoes [34]. Like *Bt*, the *Bb*-Cyt2Ba strain will increase its efficiency in mosquito larvae control with a suitable formulation, which is helpful to the development of *Bb*-Cyt2Ba strain as a commercial product.

In addition, the effects of *Bb*-Cyt2Ba and WT infection on mosquito fecundity were also assessed in our study. *Bb*-Cyt2Ba infection resulted in a dramatic reduction in the fecundity of target mosquitoes. Despite the LT_50_ of *Bb*-Cyt2Ba-infected mosquitoes suggesting that death was not quick enough to completely inhibit the females from going through their gonotrophic cycles, fecundity reduction might be a byproduct of rapid fungal invasion of *Bb*-Cyt2Ba, which is hypothesized to be an adaptive strategy of the host [35]. These data suggested that strain *Bb*-Cyt2Ba has the potential to reduce the size of *Aedes* mosquito populations by severely compromising fecundity.

Compared to our previous study, the virulence of *Bb*-Cyt2Ba to *Ae*. *albopictus* mosquitoes is a little bit higher than that of the *B*. *bassiana* expressing scorpion neurotoxin AaIT toxin [14]. Due to their high specificity against insects and low toxicity to vertebrates and plants of these proteins [36–38], *AaIT* and *Cyt2Ba* genes are both good choices for introduction into the genome of *B*. *bassiana* to increase the fungal pathogenicity.

Entomopathogenic fungi show considerable potential to be developed as biopesticides [39–41]. The production and application of fungi both involve relatively simple infrastructures and processes [40, 42], which can be readily adopted in mosquito-borne disease-endemic countries. However, before gene-modified fungi can be used and integrated into control programs, more data on the environmental safety, their effect on nontarget insect hosts and the possibility of gene flow are required.

In conclusion, our data showed that expression of the *Bacillus thuringiensis* toxin Cyt2Ba in *B*. *bassiana* increased its effectiveness against two important mosquito vectors, *Ae*. *aegypti* and *Ae*. *albopictus*. The median lethal times were shorter in the mosquito adult and larval groups infected with the *Bb*-Cyt2Ba strain compared with the groups infected with the wild type strain. In addition, the fecundity of the females was dramatically reduced by *Bb*-Cyt2Ba infection compared with WT infection. This recombinant *B*. *bassiana* strain *Bb*-Cyt2Ba was valuable in development as a bioinsecticide for mosquito control and even for other types of pest control.

## Supporting information

S1 Fig Identification of the genetic stability of the *Cyt2Ba* gene in the different generations of recombinant *Beauveria bassiana*. Conventional PCR was performed for the *bar* (A) and *Cyt2Ba* (B) genes with the genomic DNA of the different generations of the transformant *Bb*-Cyt2Ba and the WT. M, DNA maker; lane 1, the positive control; lane 2, the negative control (WT); lanes 3–5, the transformants that were subcultured for 1 to 3 generations on CDA with 150 μg/ml PPT; lane 6, the transformants subcultured on CDA with 400 μg/ml PPT.
(TIF)

S1 Table Results of the log-rank test for the different concentrations of *Bb*-Cyt2Ba or the WT against *Aedes* mosquitoes (larvae or adults). (DOCX)

S2 Table Results of the log-rank test on the different fungal strains *Bb*-Cyt2Ba and WT against *Aedes* mosquitoes (larvae or adults) at each given concentration. (DOCX)

S3 Table Analysis of the effects of the different fungal strains on the oviposition rate of female mosquitoes by the cross-tabulation analyses. (DOCX)
