# Supplementary figures and images for: Expression of Bacillus thuringiensis toxin Cyt2Ba in the entomopathogenic fungus Beauveria bassiana increases its virulence towards Aedes mosquitoes
Source: PLoS Negl Trop Dis. 2019 Jul 15;13(7):e0007590. doi: 10.1371/journal.pntd.0007590 (PMC6667155; doi:10.1371/journal.pntd.0007590)

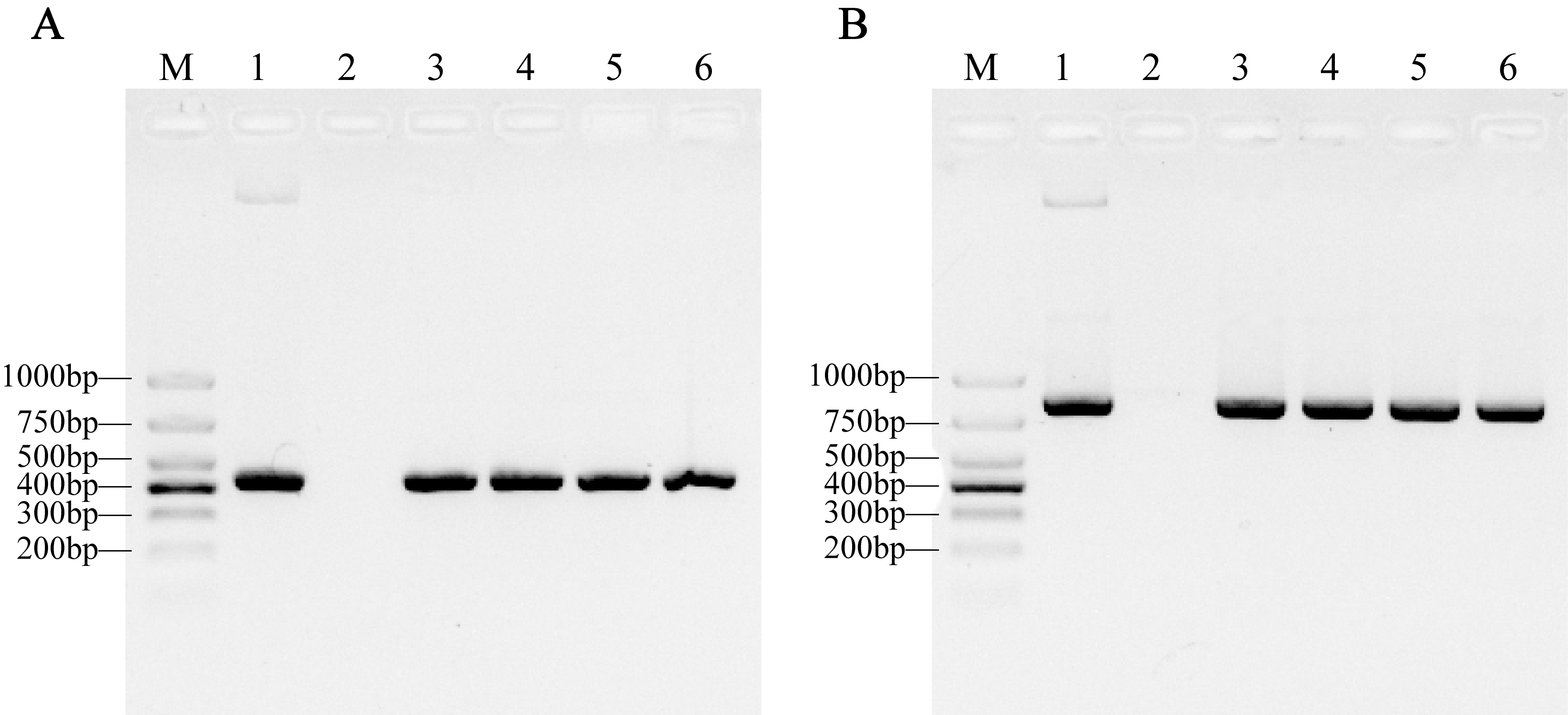

Supplement: S1 Fig — Conventional PCR was performed for the bar (A) and Cyt2Ba (B) genes with the genomic DNA of the different generations of the transformant Bb-Cyt2Ba and the WT. M, DNA maker; lane 1, the positive control; lane 2, the negative control (WT); lanes 3–5, the transformants that were subcultured for 1 to 3 generations on CDA with 150 μg/ml PPT; lane 6, the transformants subcultured on CDA with 400 μg/ml PPT. (TIF) [file pntd.0007590.s001.tif]
