# Supplementary material for: Expression of Bacillus thuringiensis toxin Cyt2Ba in the entomopathogenic fungus Beauveria bassiana increases its virulence towards Aedes mosquitoes
Source: PLoS Negl Trop Dis. 2019 Jul 15;13(7):e0007590. doi: 10.1371/journal.pntd.0007590 (PMC6667155; doi:10.1371/journal.pntd.0007590)
Supplement: S1 Table — (DOCX) [file pntd.0007590.s002.docx]

**S1 Table Results of the log-rank test for the different concentrations of *Bb*-Cyt2Ba or the WT against *Aedes* mosquitoes (larvae or adults)**

| **Mosquitoes** | **Fungal strains** | **χ^2^** | **Df** | ***P*** |
| --- | --- | --- | --- | --- |
| *Ae. aegypti* adults | *Bb*-Cyt2Ba | 156.437 | 2 | <0.001 |
|  | WT | 132.229 | 2 | <0.001 |
| *Ae. aegypti* larvae | *Bb*-Cyt2Ba | 105.839 | 2 | <0.001 |
|  | WT | 136.668 | 2 | <0.001 |
| *Ae. albopictus* adults | *Bb*-Cyt2Ba | 114.755 | 2 | <0.001 |
|  | WT | 143.622 | 2 | <0.001 |
| *Ae. albopictus* larvae | *Bb*-Cyt2Ba | 113.128 | 2 | <0.001 |
|  | WT | 153.191 | 2 | <0.001 |

*P* < 0.05 means that the difference is significant.
