# Supplementary material for: Expression of Bacillus thuringiensis toxin Cyt2Ba in the entomopathogenic fungus Beauveria bassiana increases its virulence towards Aedes mosquitoes
Source: PLoS Negl Trop Dis. 2019 Jul 15;13(7):e0007590. doi: 10.1371/journal.pntd.0007590 (PMC6667155; doi:10.1371/journal.pntd.0007590)
Supplement: S2 Table — (DOCX) [file pntd.0007590.s003.docx]

**S2 Table Results of the log-rank test on the different fungal strains *Bb*-Cyt2Ba and WT against *Aedes* mosquitoes (larvae or adults) at each given concentration**

| **Mosquitoes** | **Concentrations (conidia/ml)** | **χ^2^** | **Df** | ***P*** |
| --- | --- | --- | --- | --- |
| *Ae. aegypti* adults | 1 x 10^8^ | 44.708 | 1 | <0.001 |
|  | 1 x 10^7^ | 32.720 | 1 | <0.001 |
|  | 1 x 10^6^ | 62.985 | 1 | <0.001 |
| *Ae. aegypti* larvae | 1 x 10^7^ | 54.678 | 1 | <0.001 |
|  | 1 x 10^6^ | 7.869 | 1 | 0.005 |
|  | 1 x 10^5^ | 2.079 | 1 | 0.149 |
| *Ae. albopictus* adults | 1 x 10^8^ | 21.222 | 1 | <0.001 |
|  | 1 x 10^7^ | 22.737 | 1 | <0.001 |
|  | 1 x 10^6^ | 17.763 | 1 | <0.001 |
| *Ae. albopictus* larvae | 1 x 10^7^ | 25.020 | 1 | <0.001 |
|  | 1 x 10^6^ | 25.671 | 1 | <0.001 |
|  | 1 x 10^5^ | 15.605 | 1 | <0.001 |

*P* < 0.05 means that the difference is significant.
