# Supplementary material for: Expression of Bacillus thuringiensis toxin Cyt2Ba in the entomopathogenic fungus Beauveria bassiana increases its virulence towards Aedes mosquitoes
Source: PLoS Negl Trop Dis. 2019 Jul 15;13(7):e0007590. doi: 10.1371/journal.pntd.0007590 (PMC6667155; doi:10.1371/journal.pntd.0007590)
Supplement: S3 Table — (DOCX) [file pntd.0007590.s004.docx]

**S3 Table Analysis of the effects of the different fungal strains on the oviposition rate of female mosquitoes by the cross-tabulation analyses.**

| **Mosquitoes** | **Fungal strains** | **χ^2^** | **Df** | ***P*** |
| --- | --- | --- | --- | --- |
| *Ae. aegypti* | *Bb*-Cyt2Ba VS WT | 4.848 | 1 | 0.028 |
|  | *Bb*-Cyt2Ba VS Control | 35.714 | 1 | <0.001 |
|  | WT VS Control | 16.875 | 1 | <0.001 |
| *Ae. albopictus* | *Bb*-Cyt2Ba VS WT | 5.000 | 1 | 0.025 |
|  | *Bb*-Cyt2Ba VS Control | 33.409 | 1 | <0.001 |
|  | WT VS Control | 15.360 | 1 | <0.001 |

*P* < 0.05 means that the difference is significant.
